# Supplementary figures and images for: Rab7-dependent regulation of goblet cell protein CLCA1 modulates gastrointestinal homeostasis
Source: eLife. 2024 Apr 9;12:RP89776. doi: 10.7554/eLife.89776 (PMC11003743; doi:10.7554/eLife.89776)

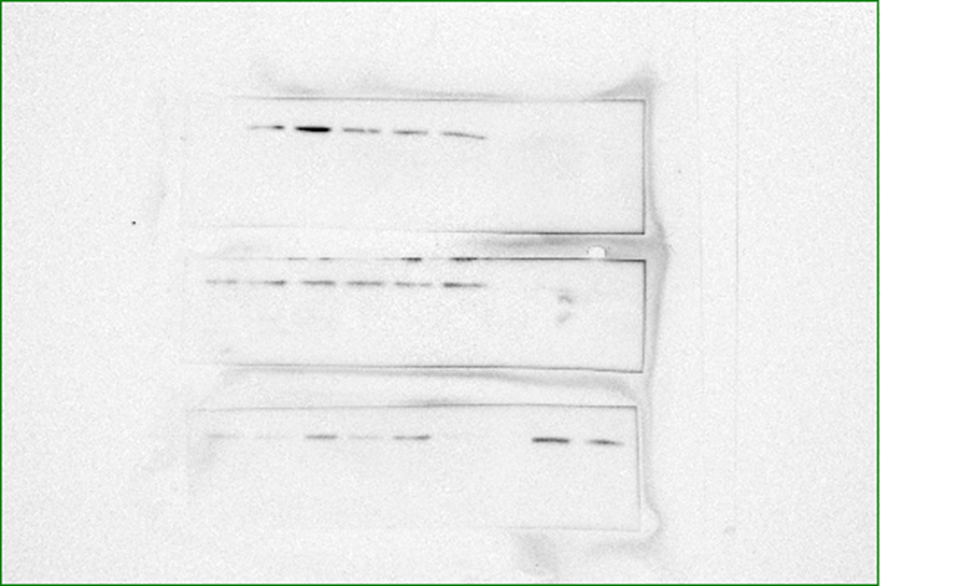

Supplement: Figure 1—source data 1. [file elife-89776-fig1-data1.zip › Figure 1- source data 1.1.tif]

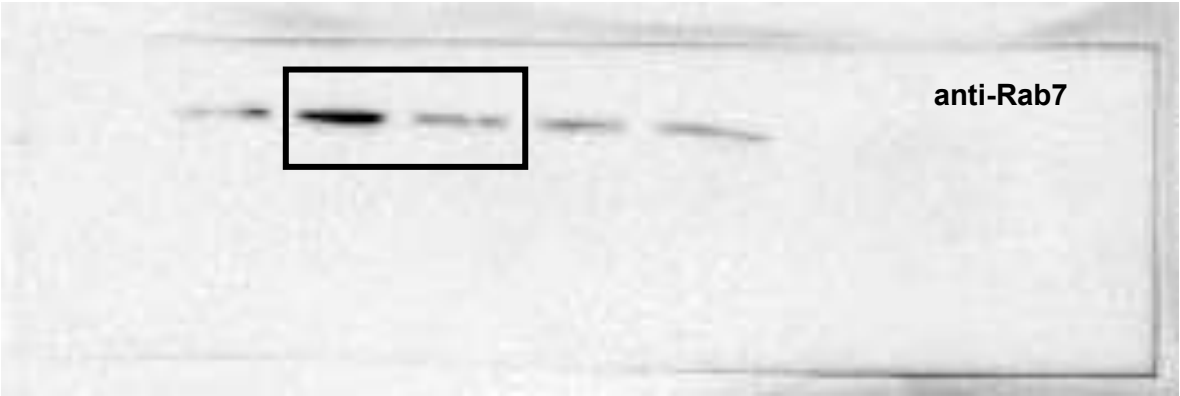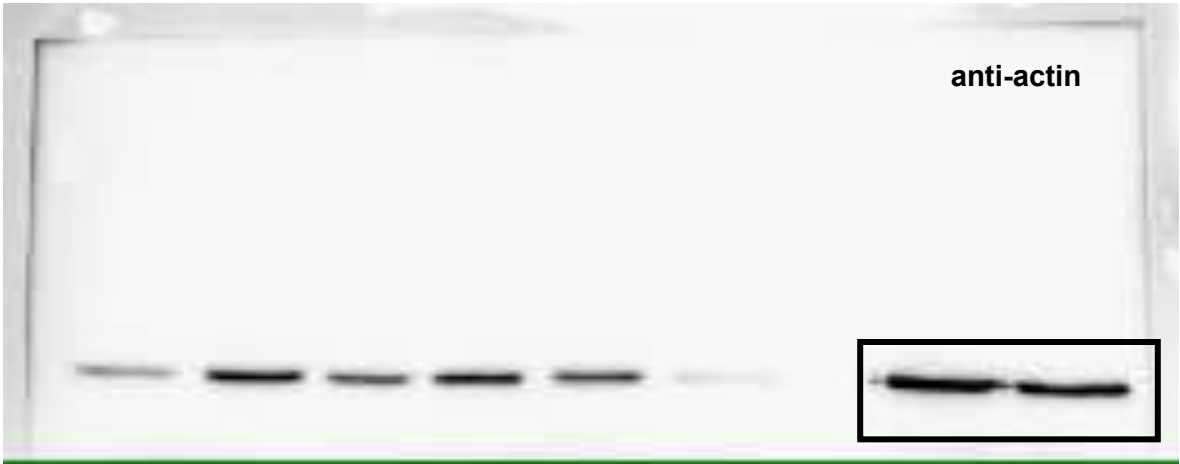

Supplement: Figure 1—source data 1. [file elife-89776-fig1-data1.zip › Figure 1- source data 1.pdf]

**anti-Rab7**

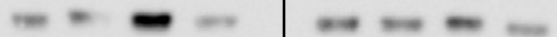

**anti-actin**

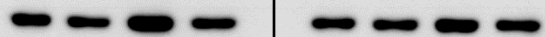

Supplement: Figure 1—source data 1. [file elife-89776-fig1-data1.zip › Figure 1- source data 2.pdf]

anti-Rab7

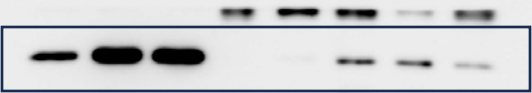

anti-GAPDH

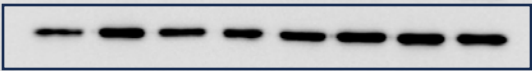

Supplement: Figure 1—source data 1. [file elife-89776-fig1-data1.zip › Figure 1- source data 3.pdf]

anti-Rab7

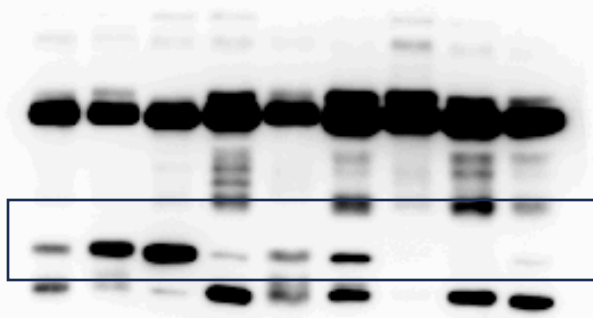

anti-GAPDH

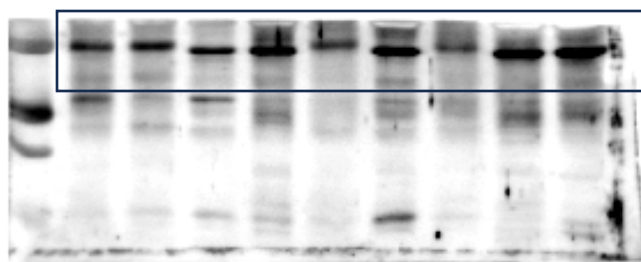

Supplement: Figure 1—source data 1. [file elife-89776-fig1-data1.zip › Figure 1- source data 4.pdf]

**anti-Rab7**

**Whole tissue lysate**

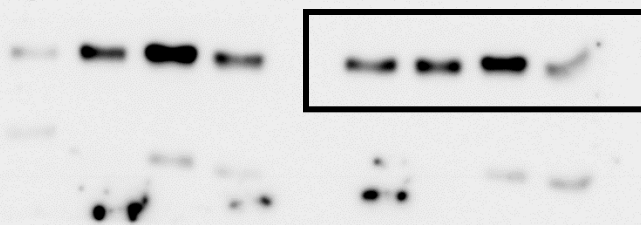

**anti-actin**

**Whole tissue lysate**

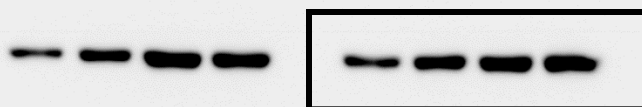

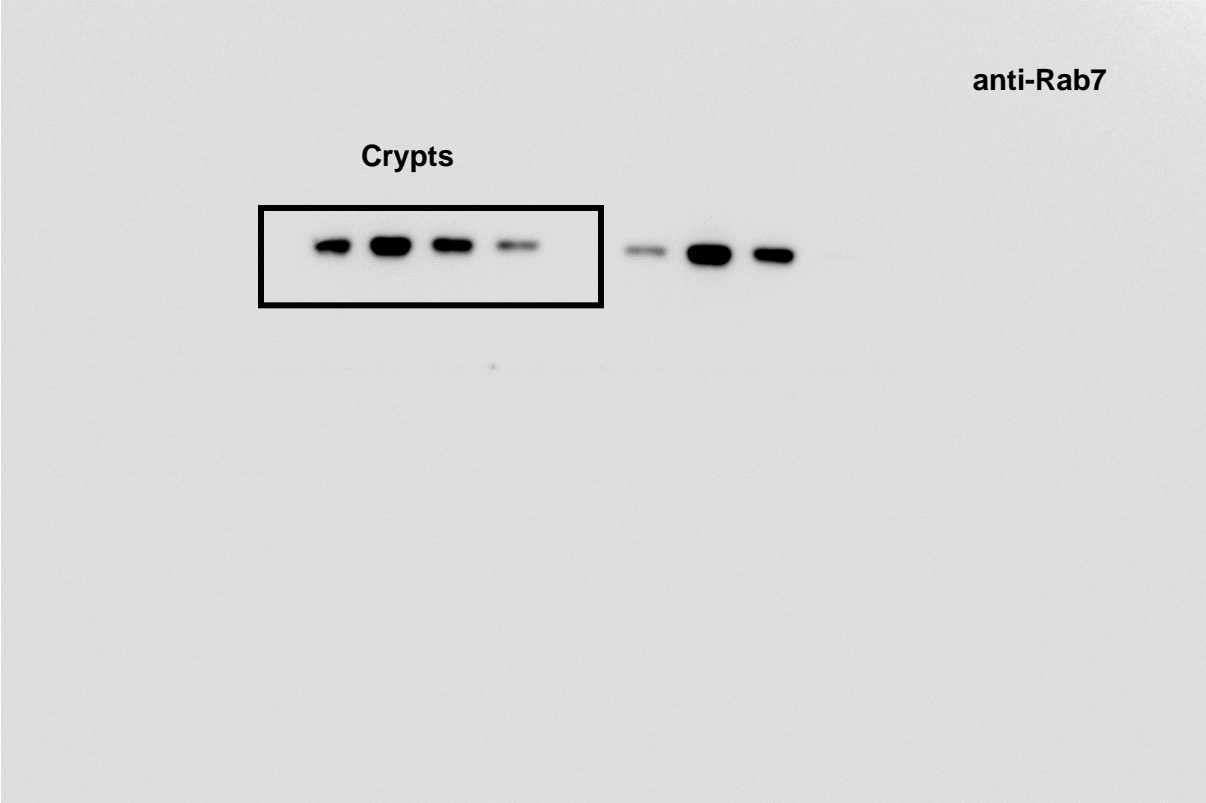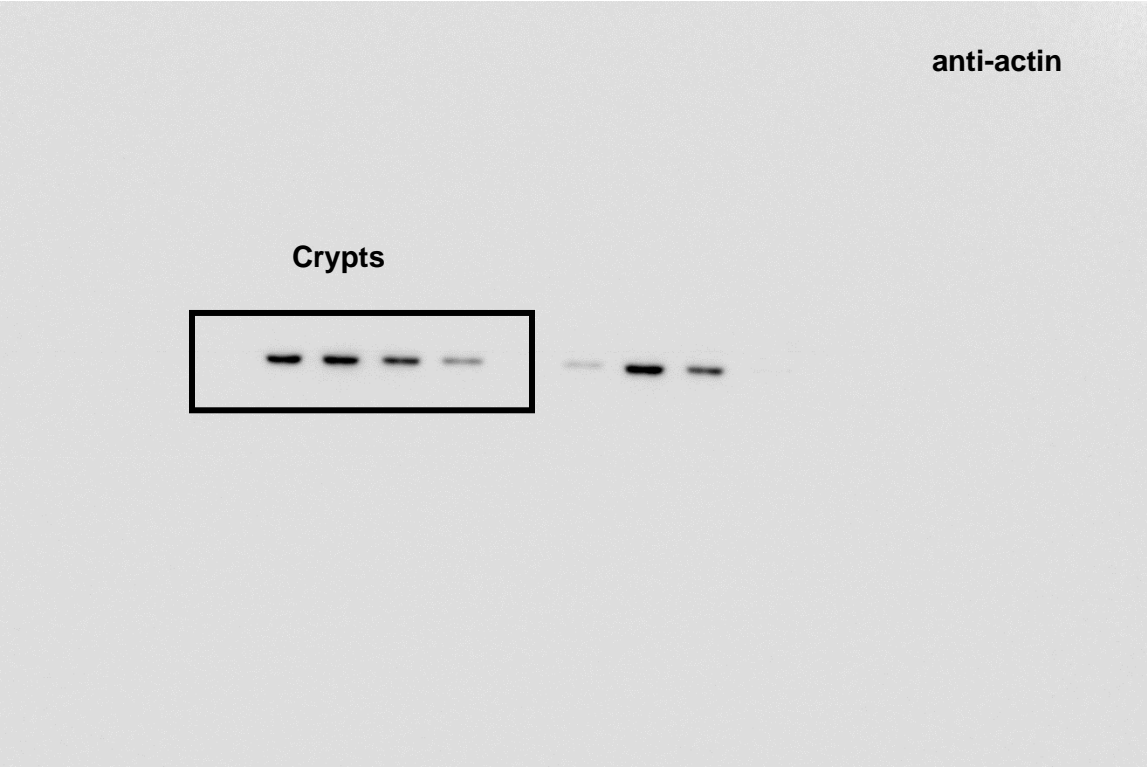

Supplement: Figure 1—figure supplement 1—source data 1. [file elife-89776-fig1-figsupp1-data1.zip › Figure 1- figure supplement 1-source data 1.pdf]

**anti-Rab7**

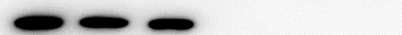

**anti-GAPDH**

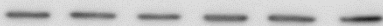

Supplement: Figure 1—figure supplement 1—source data 1. [file elife-89776-fig1-figsupp1-data1.zip › Figure 1- figure supplement 1-source data 2.pdf]

**anti-Rab7**

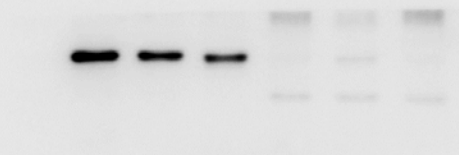

**anti-GAPDH**

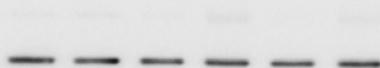

Supplement: Figure 1—figure supplement 1—source data 1. [file elife-89776-fig1-figsupp1-data1.zip › Figure 1- figure supplement 1-source data 3.pdf]

**anti-Rab7**

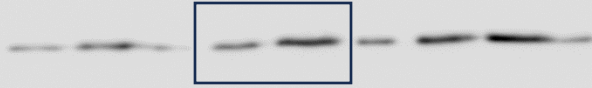

**anti-actin**

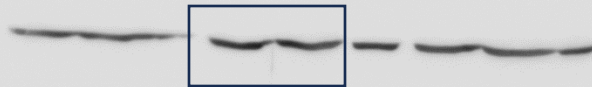

Supplement: Figure 2—figure supplement 1—source data 1. [file elife-89776-fig2-figsupp1-data1.zip › Figure 2- figure supplement 1- source data 1.pdf]

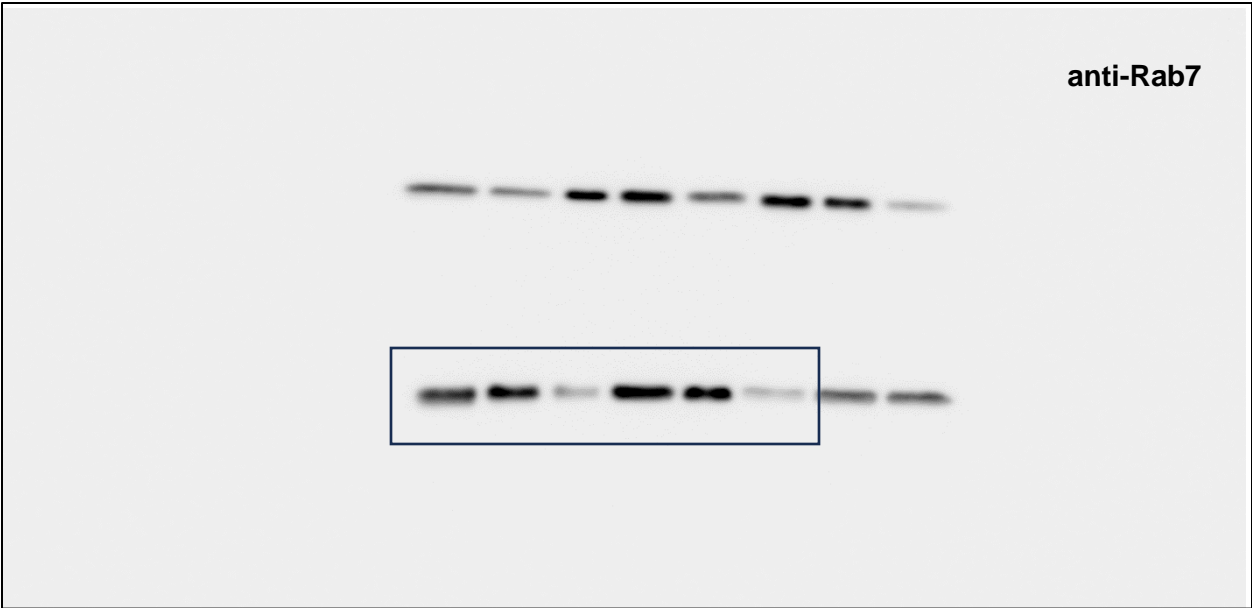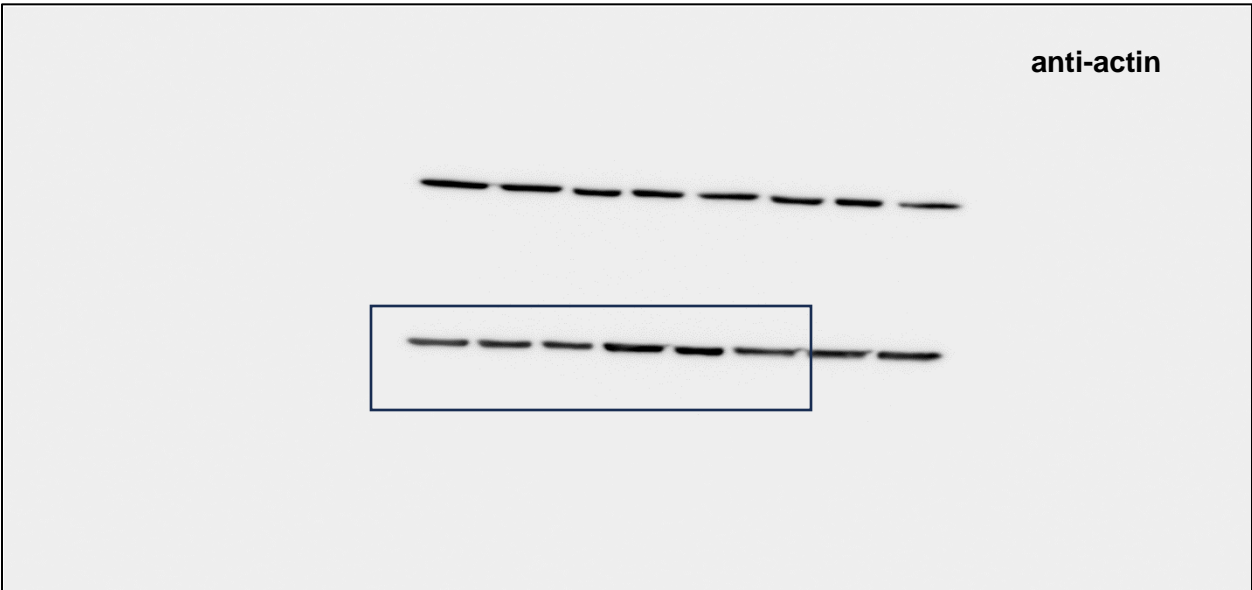

Supplement: Figure 3—source data 1. [file elife-89776-fig3-data1.zip › Figure 3- source data 1.pdf]

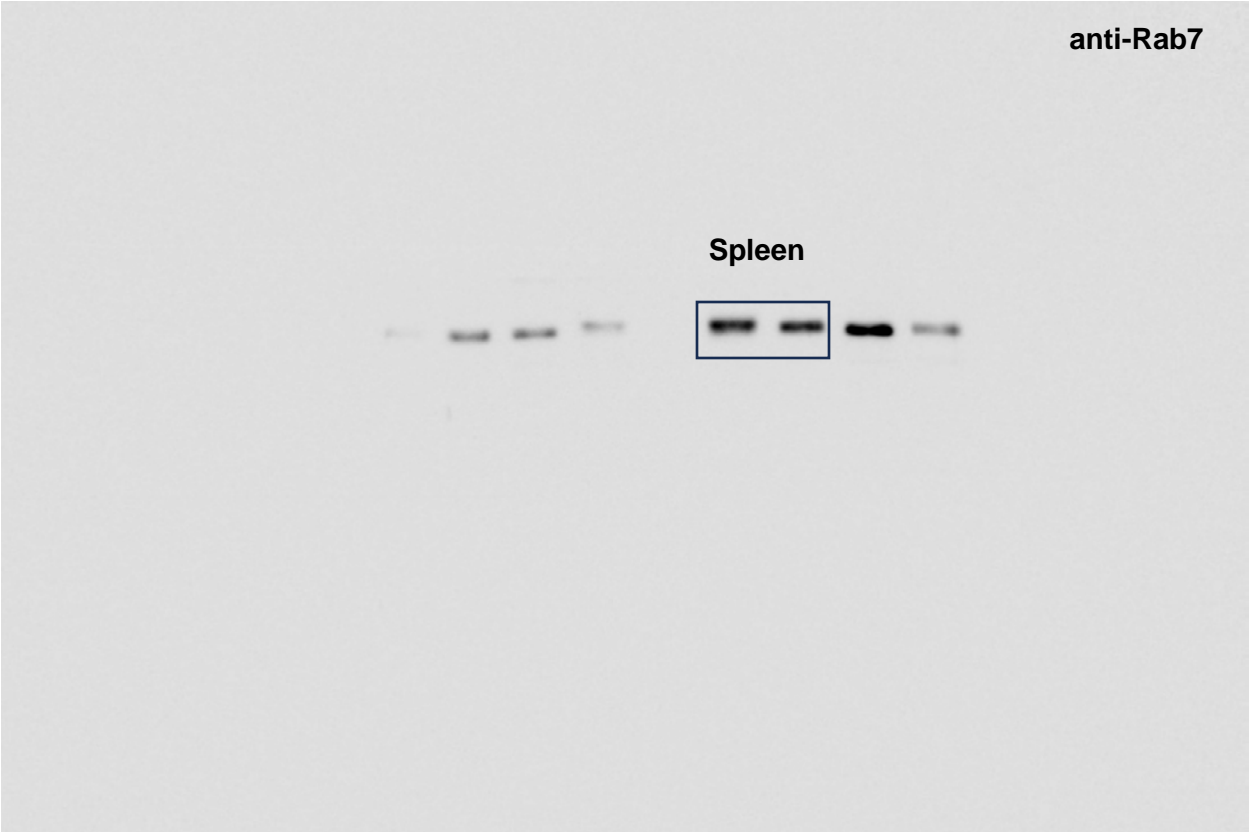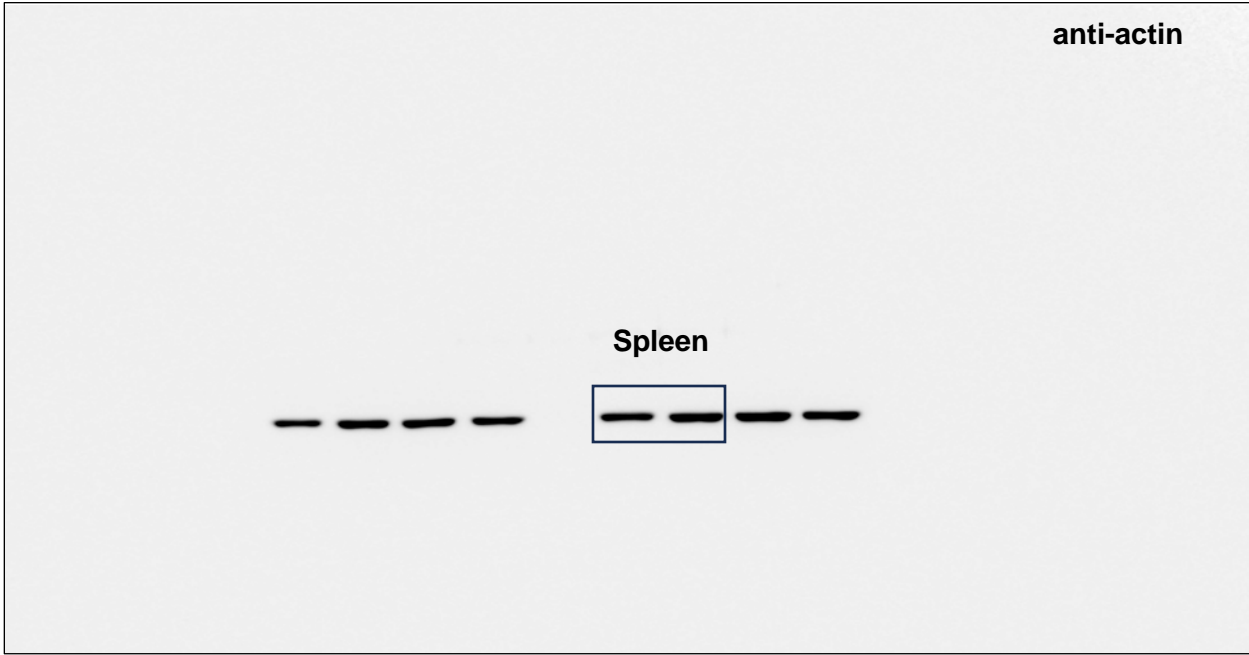

anti-Rab7

MLN

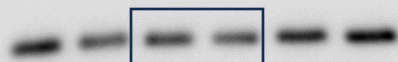

anti-actin

MLN

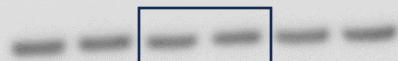

**anti-Rab7**

**Liver**

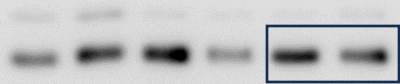

**anti-actin**

**Liver**

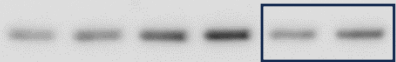

Supplement: Figure 3—figure supplement 1—source data 1. [file elife-89776-fig3-figsupp1-data1.zip › Figure 3- figure supplement 1- source data 1.pdf]

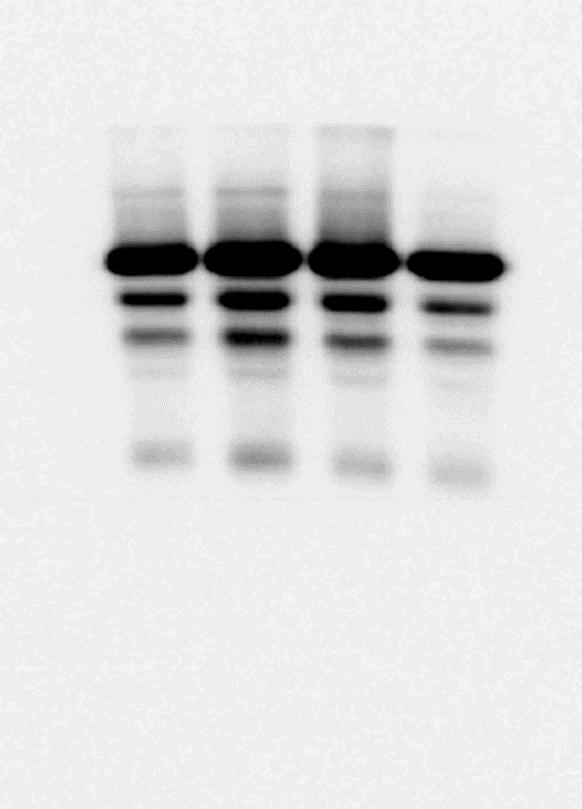

Supplement: Figure 7—source data 1. [file elife-89776-fig7-data1.zip › Figure 7- source data 1.1.tif]

anti-CLCA1

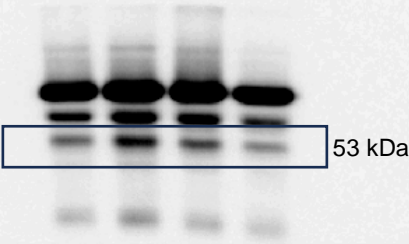

Supplement: Figure 7—source data 1. [file elife-89776-fig7-data1.zip › Figure 7- source data 1.pdf]

**anti-CLCA1**

**Tissue**

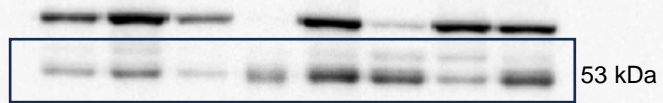

**anti-CLCA1**

**Mucus**

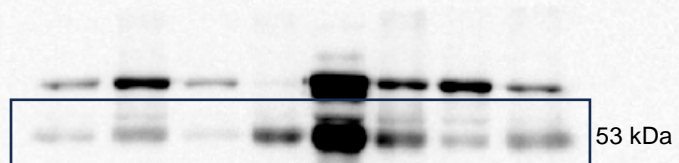

Supplement: Figure 7—source data 1. [file elife-89776-fig7-data1.zip › Figure 7- source data 2.pdf]

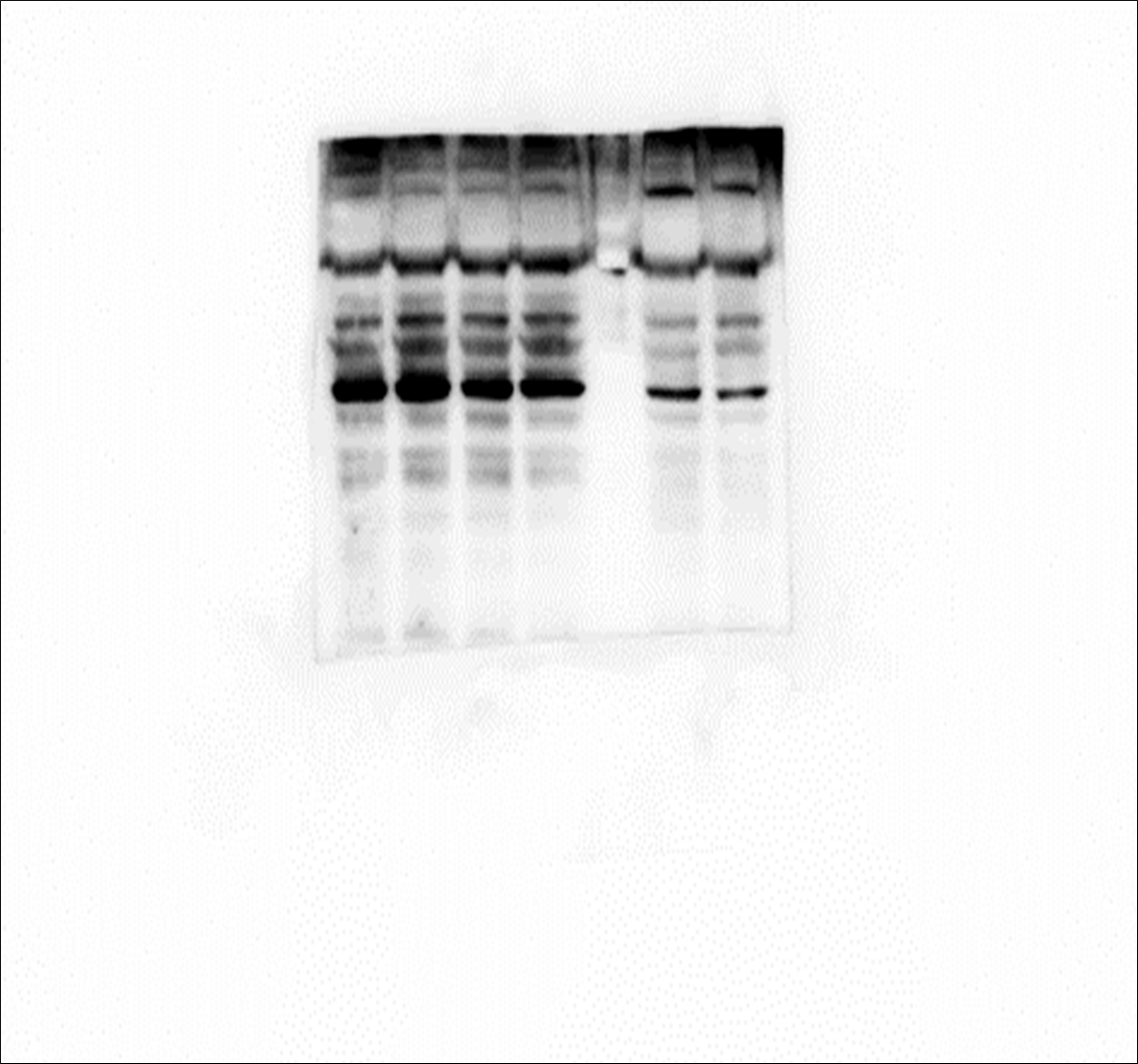

Supplement: Figure 7—source data 1. [file elife-89776-fig7-data1.zip › Figure 7- source data 3.1.tif]

**anti-CLCA1**

53 kDa

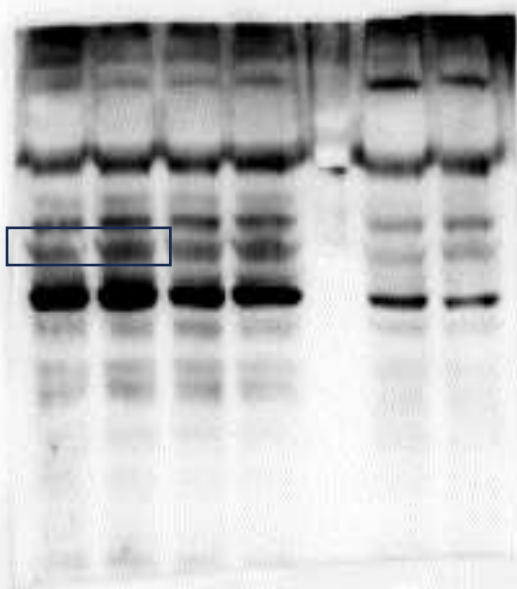

**anti-actin**

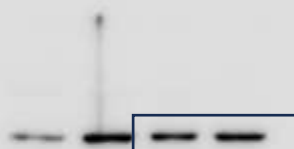

Supplement: Figure 7—source data 1. [file elife-89776-fig7-data1.zip › Figure 7- source data 3.pdf]

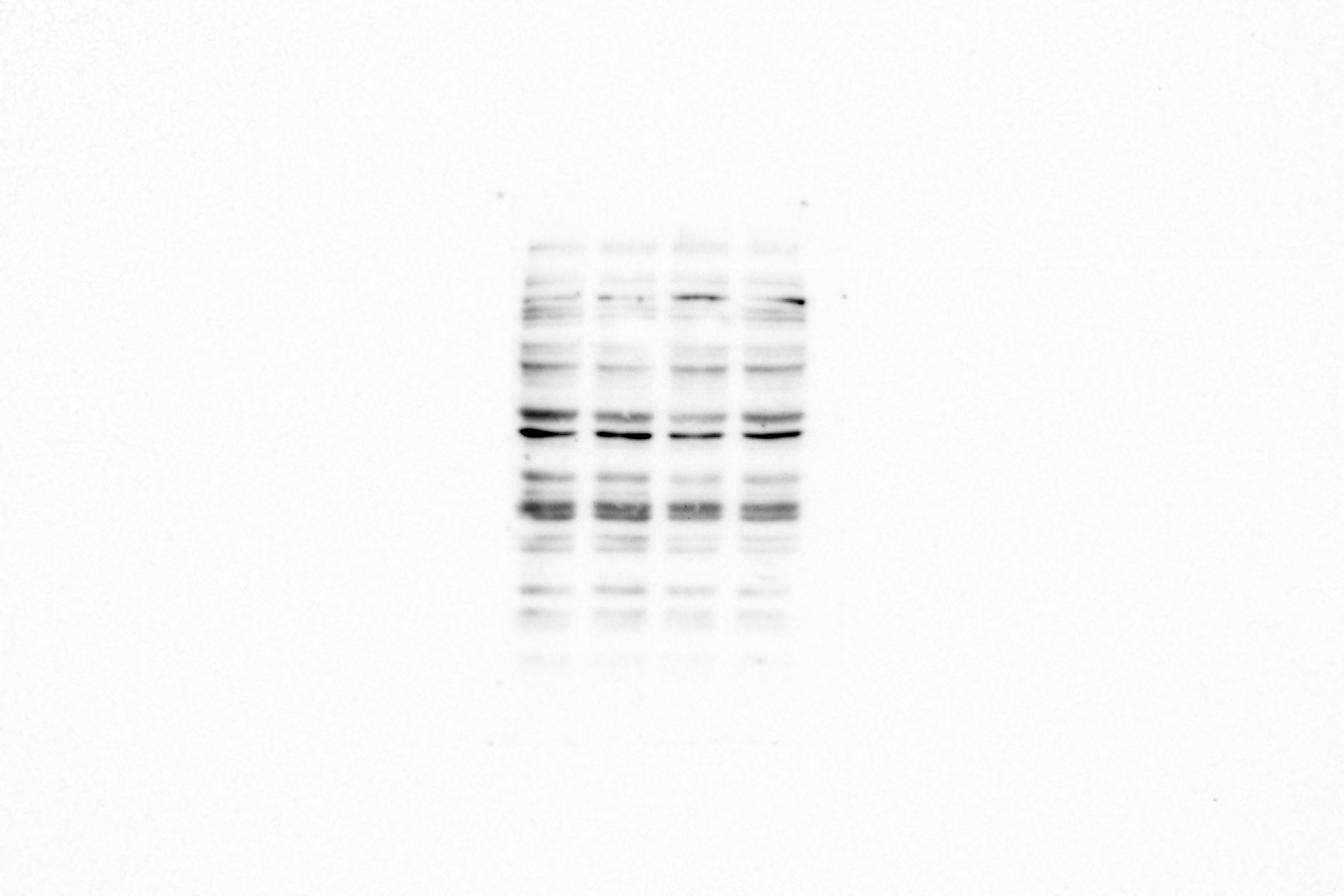

Supplement: Figure 7—source data 1. [file elife-89776-fig7-data1.zip › Figure 7- source data 4.1.tif]

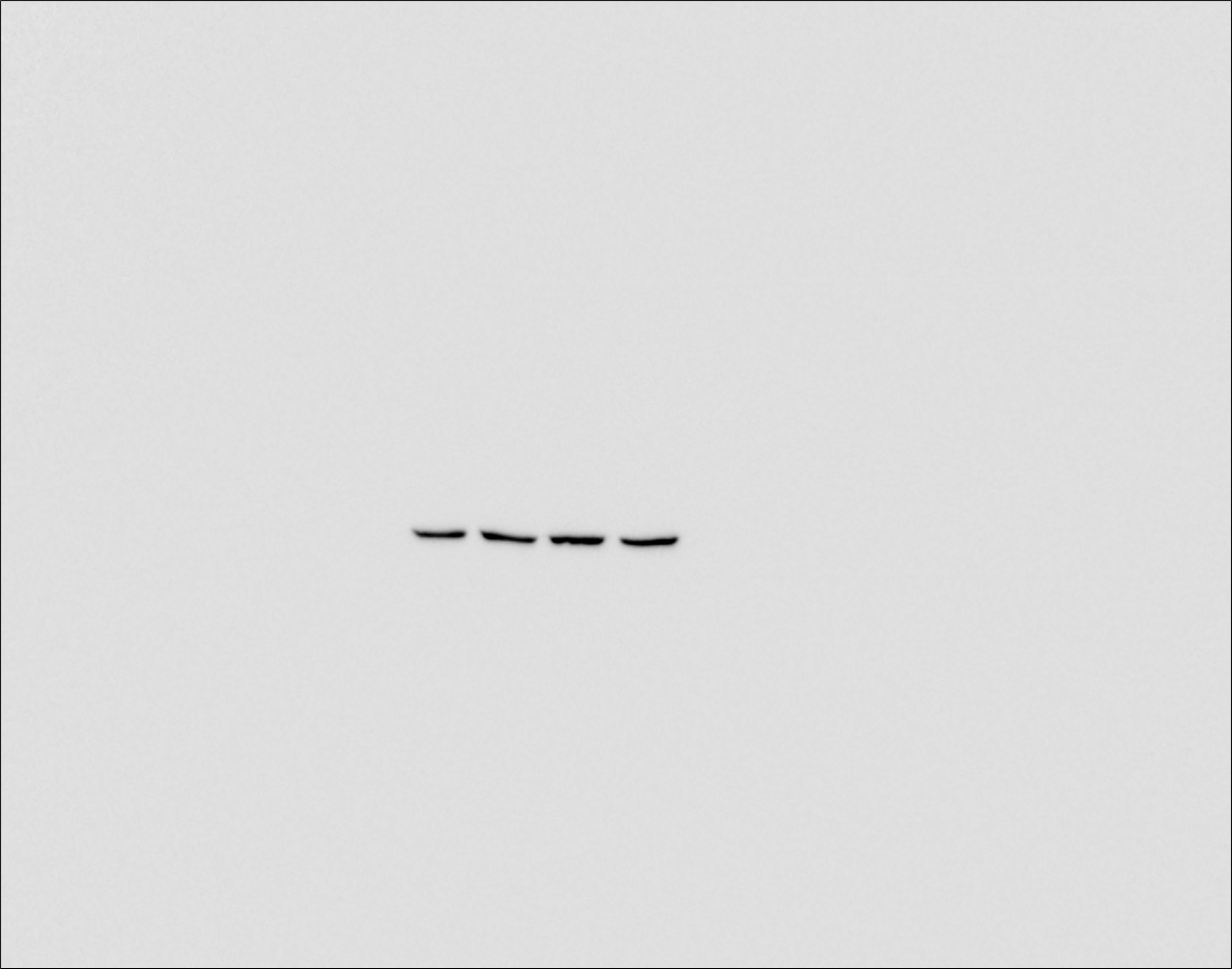

Supplement: Figure 7—source data 1. [file elife-89776-fig7-data1.zip › Figure 7- source data 4.2.tif]

anti-CLCA1

53 kDa

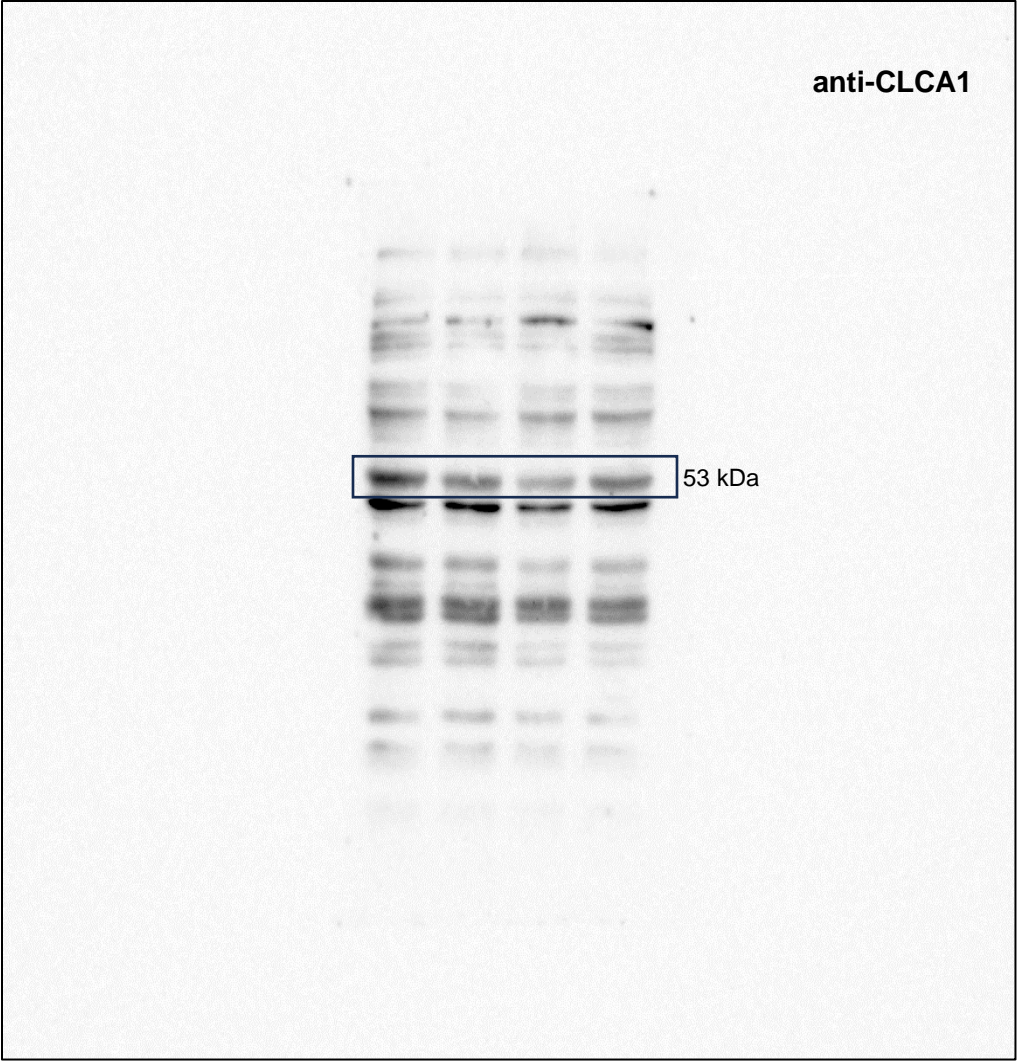

**anti-actin**

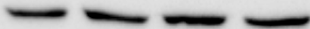

Supplement: Figure 7—source data 1. [file elife-89776-fig7-data1.zip › Figure 7- source data 4.pdf]

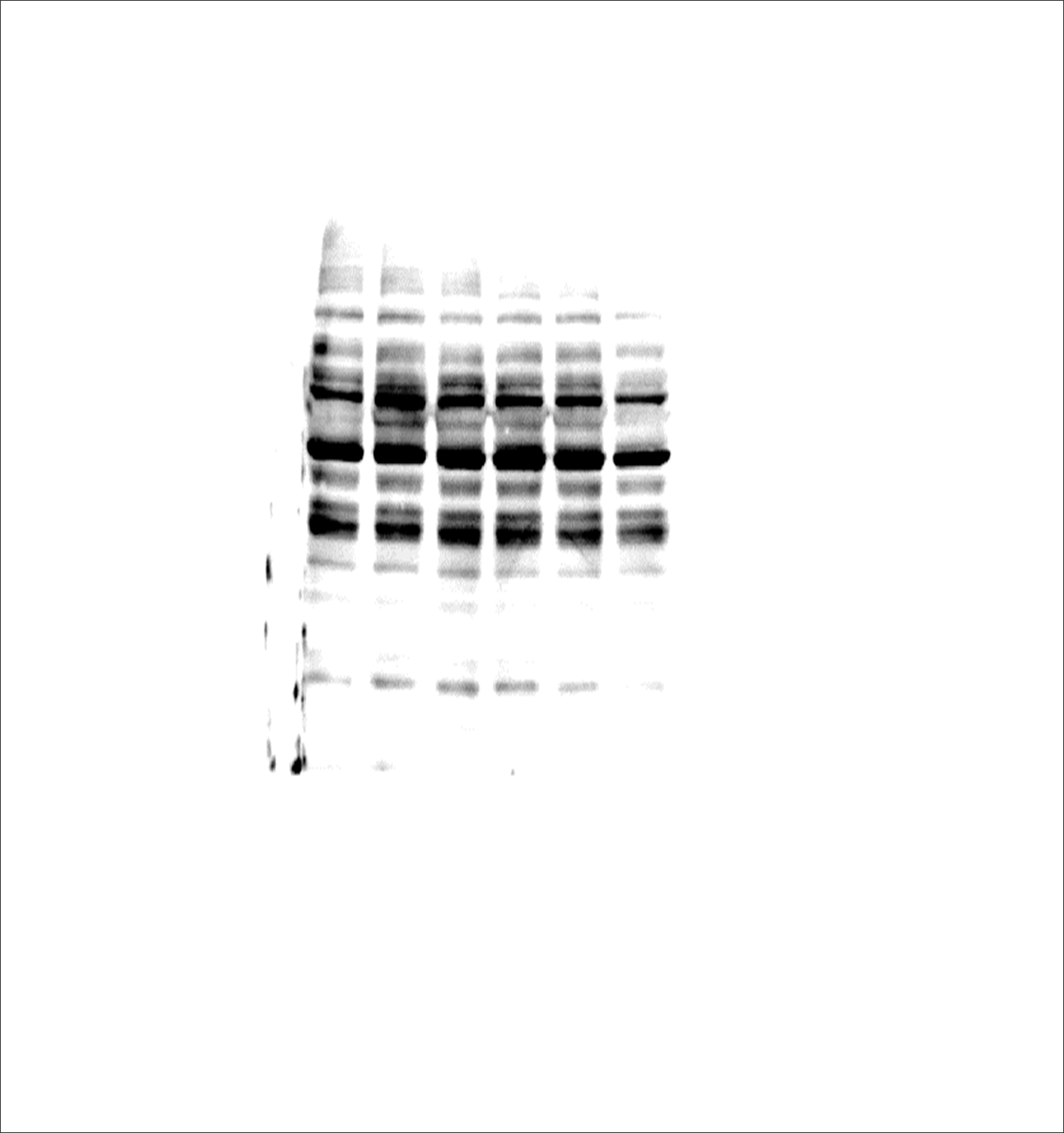

Supplement: Figure 7—source data 1. [file elife-89776-fig7-data1.zip › Figure 7- source data 5.1.tif]

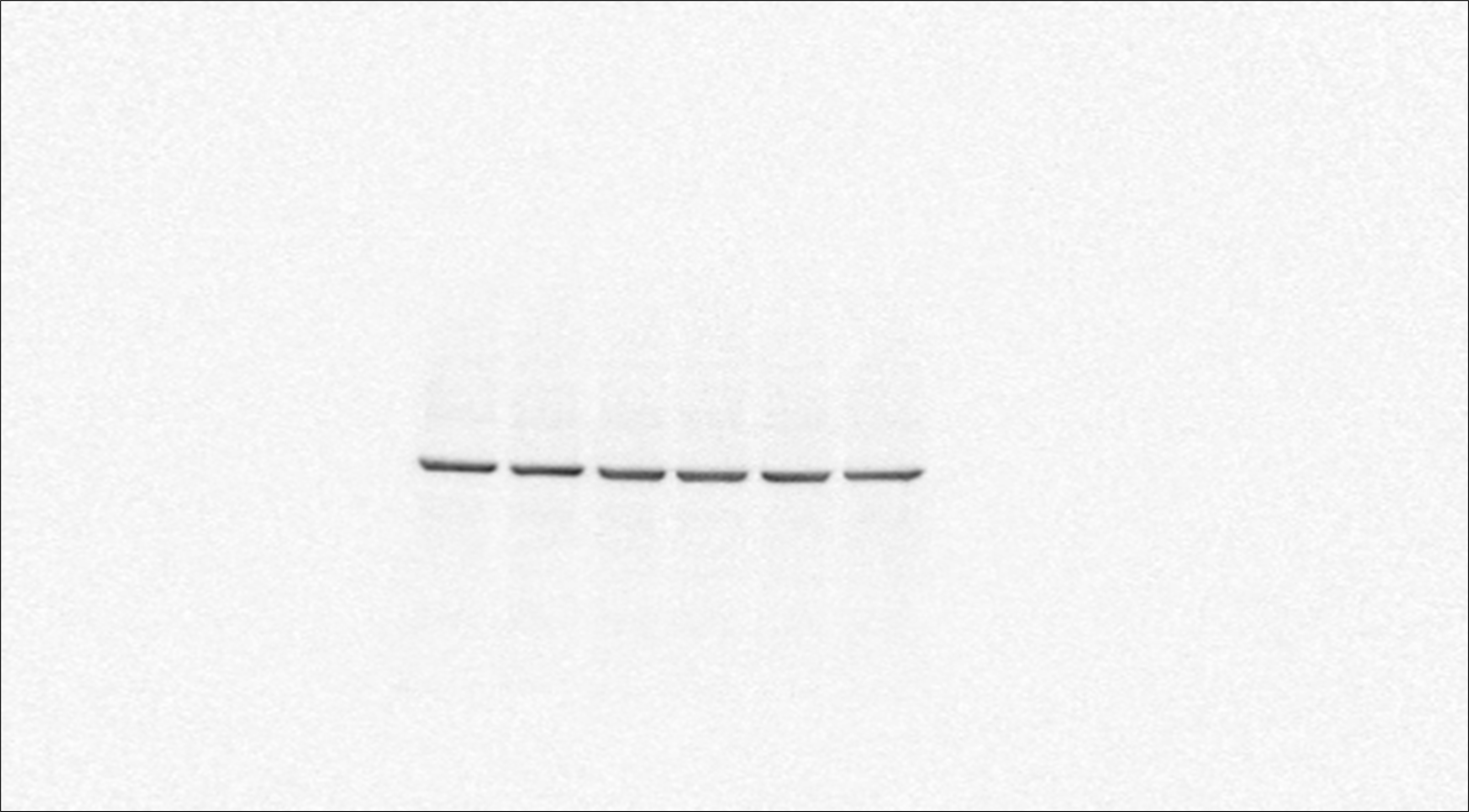

Supplement: Figure 7—source data 1. [file elife-89776-fig7-data1.zip › Figure 7- source data 5.2.tif]

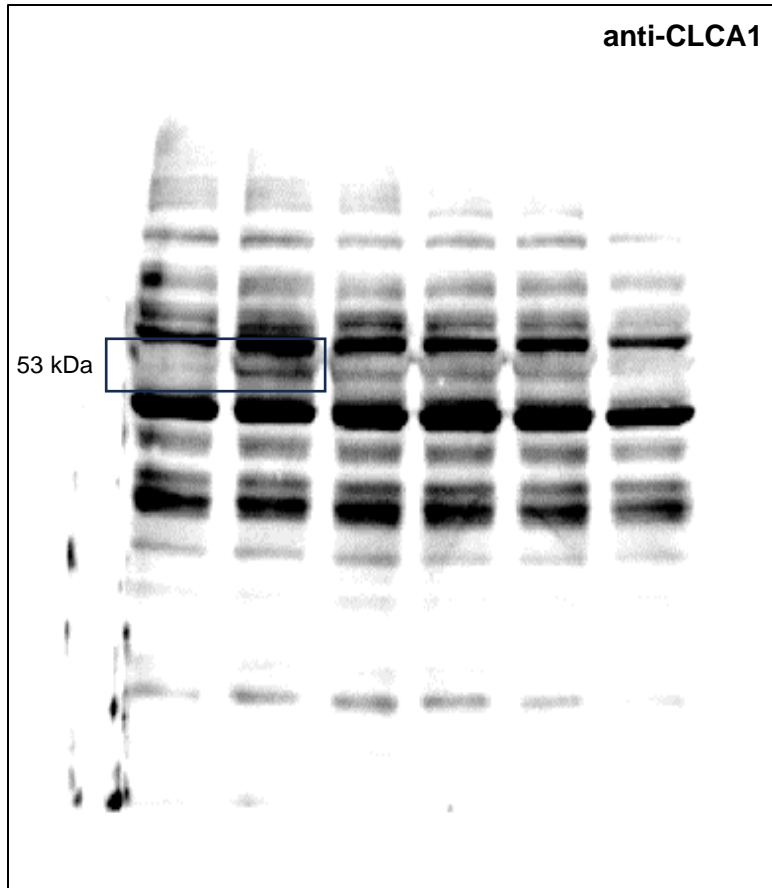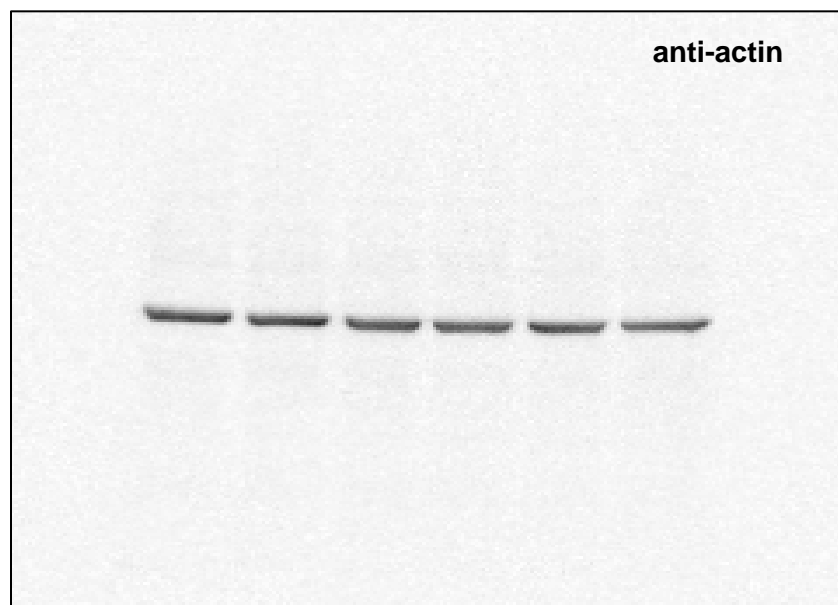

Supplement: Figure 7—source data 1. [file elife-89776-fig7-data1.zip › Figure 7- source data 5.pdf]

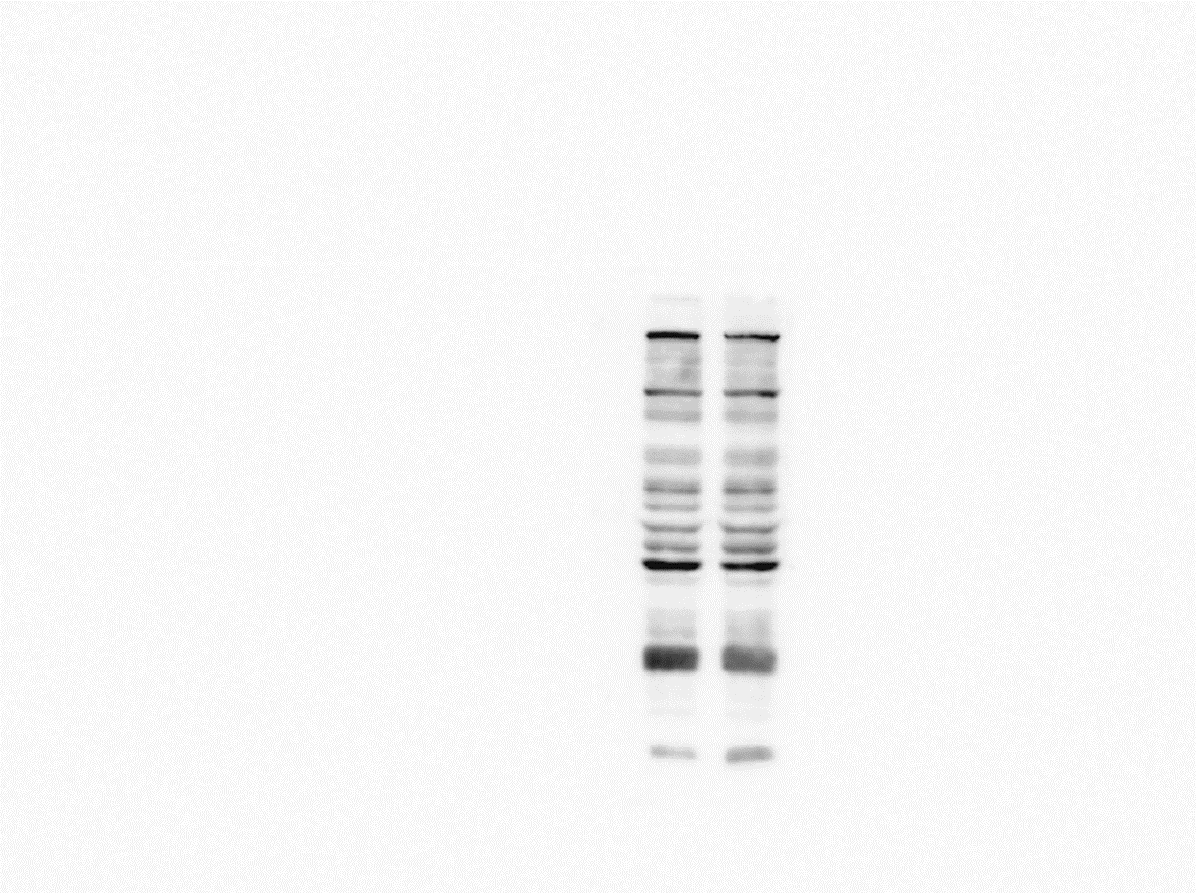

Supplement: Figure 7—figure supplement 1—source data 1. [file elife-89776-fig7-figsupp1-data1.zip › Figure 7- figure supplement 1- source data 1.1.tif]

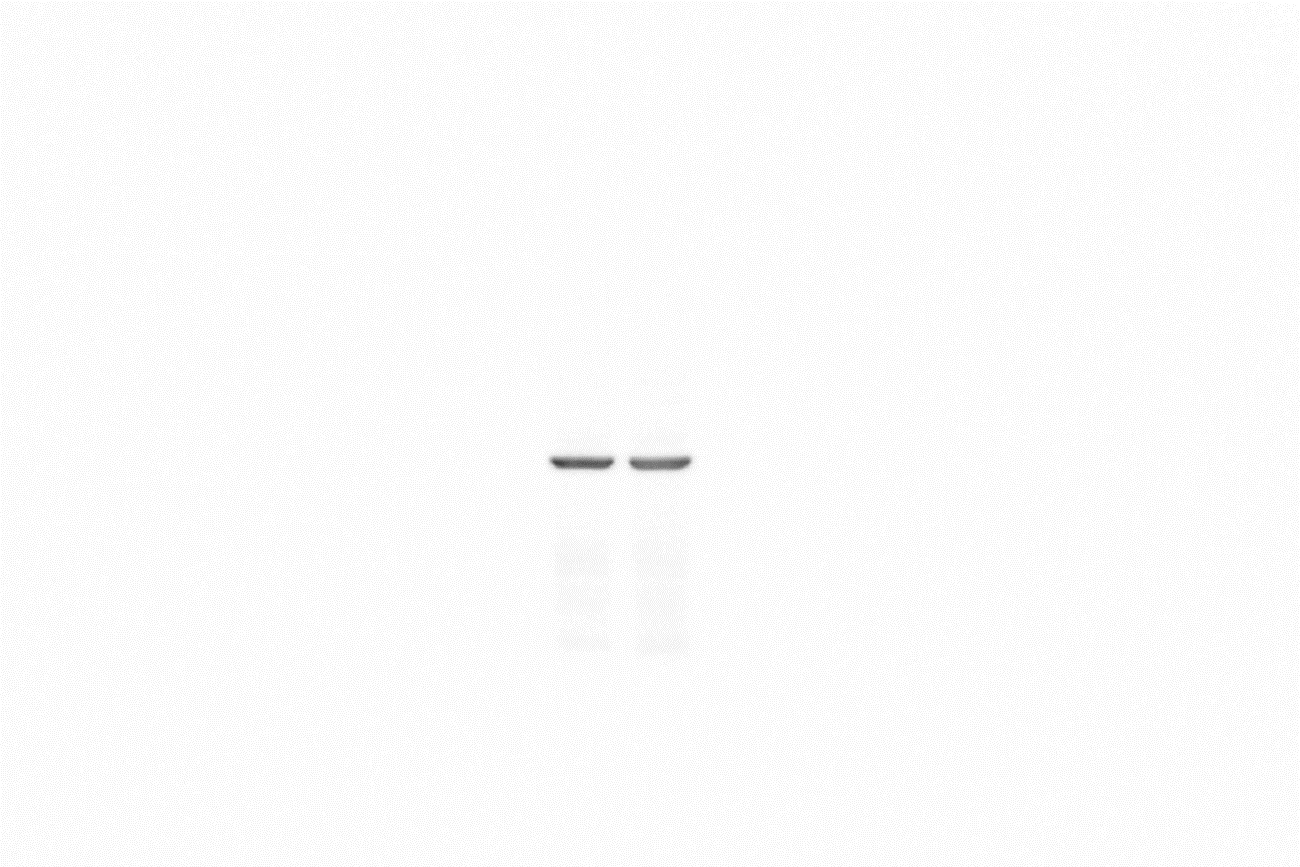

Supplement: Figure 7—figure supplement 1—source data 1. [file elife-89776-fig7-figsupp1-data1.zip › Figure 7- figure supplement 1- source data 1.2.tif]

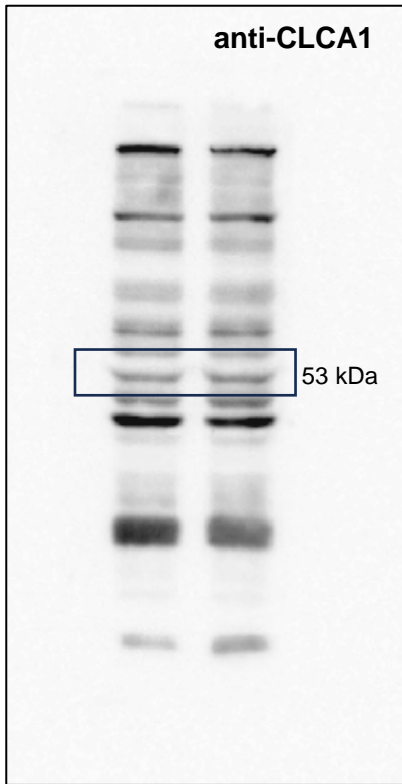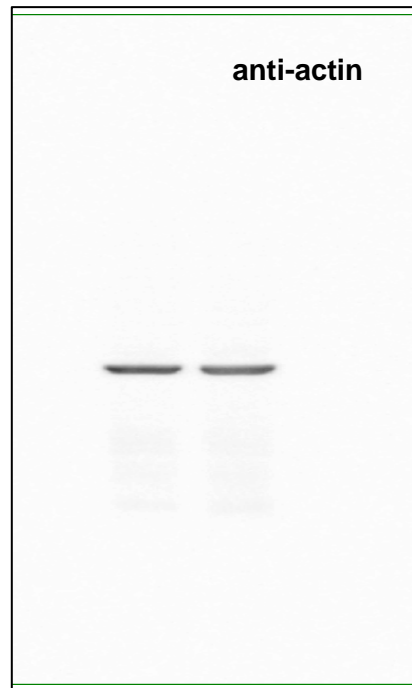

Supplement: Figure 7—figure supplement 1—source data 1. [file elife-89776-fig7-figsupp1-data1.zip › Figure 7- figure supplement 1- source data 1.pdf]

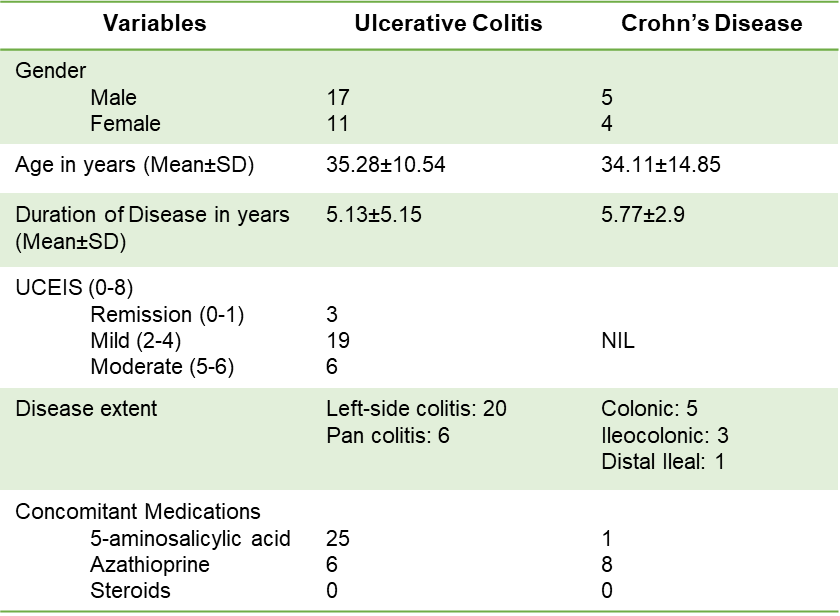

Supplement: Supplementary file 1. [file elife-89776-supp1.docx]
