# Supplementary material for: Rab7-dependent regulation of goblet cell protein CLCA1 modulates gastrointestinal homeostasis
Source: eLife. 2024 Apr 9;12:RP89776. doi: 10.7554/eLife.89776 (PMC11003743; doi:10.7554/eLife.89776)
Supplement: Figure 7—figure supplement 1—source data 1. [file elife-89776-fig7-figsupp1-data1.zip › Figure 7-figure supplement 1- source data legends.docx]

**Figure 7- figure supplement 1-source data 1.1:** Original file for the western blot in Figure 7- figure supplement 1B (anti-CLCA1)

**Figure 7- figure supplement 1-source data 1.2:** Original file for the western blot in Figure 7- figure supplement 1B (anti-actin)

**Figure 7- figure supplement 1-source data 1:** Western blots labelled with relevant bands analyzed in Figure 7- figure supplement 1B (anti-CLCA1 and anti-actin)
