# Supplementary material for: Rab7-dependent regulation of goblet cell protein CLCA1 modulates gastrointestinal homeostasis
Source: eLife. 2024 Apr 9;12:RP89776. doi: 10.7554/eLife.89776 (PMC11003743; doi:10.7554/eLife.89776)
Supplement: Supplementary file 2. [file elife-89776-supp2.docx]

**List of qRT-PCR Primers**

| **Gene Name** | **Forward Primer**  **(Direction 5’-3’)** | **Reverse Primer**  **(Direction 5’-3’)** |
| --- | --- | --- |
| Human Rab7 | CATCCTGGGAGATTCTGGAGTC | TGTGTCCCATATCTGCATTGTG |
| Human CLCA1 | TTTGTTCTCCAATCCCGCCA | CGGATCACTTCCCATGTGCT |
| Mouse CLCA1 | GAACAACAACGGCTATGAGGG | GCCTGAGTCACCATGTCCTT |
| Mouse APOA1 | ATTGACTCGGGACTTCTGGG | AATTCGTCCAGGTAGGGCTG |
| Mouse TFRC | TATCTTCTGGGGCTCTGGCT | CAGGGCCAACTGGTTTCTGA |
| Human Lgr5 | AAACCTCTCCAGCTGGGTAG | TTCAGCGATCGGAGGCTAAG |
| Human Muc2 | CGAAACCACGGCCACAACGT | GACCACGGCCCCGTTAAGCA |
| Human 18S | GAGGGACAAGTGGCGTTCA | CCGGACATCTAAGGGCATCA |
| Human HPRT | GCTATAAATTCTTTGCTGACCTGCTG | AATTAACTTTTATGTCCCCTGTTGACTGG |
| Mouse HPRT | CACAGGACTAGAACACCTGC | GCTGGTGAAAAGGACCTCT |
